# A Case of Infantile Epileptic Spasms Syndrome with the *SPTBN1* Mutation and Review of βII-Spectrin Variants

**DOI:** 10.3390/genes16080904

**Published:** 2025-07-29

**Authors:** Han Na Jang, Juyeon Ryu, Seung Soo Kim, Jin-Hwa Moon

**Affiliations:** 1 Department of Pediatrics, College of Medicine, Soonchunhyang University, Cheonan 31151, Republic of Korea; janghannah85@gmail.com; 2 Department of Pediatrics, College of Medicine, Kyung-Hee University, Seoul 02447, Republic of Korea; 3 College of Medicine, Soonchunhyang University, Cheonan 31151, Republic of Korea; juyeonryu86@gmail.com; 4 Department of Pediatrics, College of Medicine, Hanyang University, Seoul 04763, Republic of Korea

**Keywords:** *SPTBN1*, βII-spectrin, infantile epileptic spasms syndrome, neurodevelopmental disorder

## Abstract

**Background**: Spectrin proteins are critical cytoskeleton components that maintain cellular structure and mediate intracellular transport. Pathogenic variants in *SPTBN1*, encoding βII-spectrin, have been associated with various neurodevelopmental disorders, including developmental delay, intellectual disability, autism spectrum disorder, and epilepsy. Here we report a Korean infant with infantile epileptic spasms syndrome (IESS) and an *SPTBN1* mutation and provide a review of this mutation. **Methods:** The genomic data of the patient were analyzed by whole exome sequencing. A comprehensive literature review was conducted to identify and analyze all reported SPTBN1 variants, resulting in a dataset of 60 unique mutations associated with neurodevelopmental phenotypes. **Case Presentation**: A 10-month-old Korean female presented with IESS associated with a de novo heterozygous *SPTBN1* mutation (c.785A>T; p.Asp262Val). The patient exhibited global developmental delay, microcephaly, hypotonia, spasticity, and MRI findings of diffuse cerebral atrophy and corpus callosum hypoplasia. Electroencephalography revealed hypsarrhythmia, confirming the diagnosis of IESS. Seizures persisted despite initial treatment with vigabatrin and steroids. Genetic analysis identified a likely pathogenic variant within the calponin homology 2 (CH2) domain of *SPTBN1*. **Conclusions**: This is the first report of an association between IESS and an *SPTBN1* CH2 domain mutation in a Korean infant. This finding expands the clinical spectrum of *SPTBN1*-related disorders and suggests domain-specific effects may critically influence phenotypic severity. Further functional studies are warranted to elucidate the pathogenic mechanisms of domain-specific variants.

## 1. Introduction

Spectrins are fundamental cytoskeleton components and serve as scaffolds that anchor cytoskeletal elements to the plasma membrane and maintain cell shape, elasticity, and integrity [1]. Initially identified in erythrocytes, spectrins are now recognized as ubiquitous cytoskeletal proteins in many tissues, including those of brain, heart, and muscles. Spectrins function as α/β heterotetramers formed by specific domain interactions and possess structural features, such as spectrin repeats (SRs), actin-binding domains, EF-hand motifs, and pleckstrin homology (PH) domains, that facilitate interactions with actin, phospholipids, and numerous membrane proteins [2].

Five types of spectrin β sub-units (βI, βII, βIII, βIV, and βV) and αII spectrins in mammals have been identified [2], and of these, βII-spectrin (encoded by *SPTBN1*) has been associated with neurodevelopmental disabilities, including delayed development, epilepsy, and autism spectrum disorder (ASD) [3].

Here we report a case of infantile epilepsy spasm syndrome (IESS) in an infant with a *SPTBN1* mutation, and provide a comprehensive review of the βII-spectrin mutation.

## 2. Methods

Genomic DNA was isolated from saliva by buccal swab. All exons of all human genes were captured using a xGen Custom Hyb Panel v1 (Integrated DNA Technologies, Coralville, IA, USA) and sequenced using a NovaSeq platform (Illumina, San Diego, CA, USA). Raw genome sequences were aligned to the reference sequence (NCBI genome assembly GRCh38). The sequencing metrics are consistent with high-quality whole exome sequencing data and are considered suitable for downstream analysis.

A systematic search of the literature was conducted using PubMed (MEDLINE) for articles published in English up to July 2025. The search strategy included the following terms: “SPTBN1” OR “βII-spectrin” OR “spectrin” AND “epilepsy” OR “infantile spasms” OR “epileptic encephalopathy” OR “neurodevelopmental disorder”, “rare diseases”. Additional references were retrieved manually based on citation tracking. Duplicate records were removed, and titles/abstracts were screened. A total of 14 articles were included in the final review.

The study was conducted in accordance with the Declaration of Helsinki and approved by the Soonchunhyang Cheonan Hospital Medical Sciences Ethics Committee (IRB number 2025-05-034).

## 3. Results

A 10-month-old female visited our pediatric neurology clinic with a one-week history of sudden-onset, bilateral arm tonic extension movements. She was born as the third child to a consanguineous Korean family after an uneventful pregnancy, at 38-week gestation, weighing 2.8 kg (16th centile). Routine measurement of head circumference (HC) at birth was recorded as 34 cm (54th centile). At her initial presentation, physical examination showed her height (72 cm, 58th percentile) and body weight (9 kg, 68th percentile) were within the normal range, but she exhibited microcephaly (42 cm, <the 5th percentile). Her neurologic exam revealed increased muscle tone with right ankle spasticity and axial muscle instability without depigmented skin lesion such as hypomelanotic macule or café-au-lait spot. The Bayley Scales of Infant and Toddler Development test performed at seven months showed profound global developmental delay with the following developmental quotient (DQ) scores: cognitive DQ = 55, language DQ = 68, and motor DQ = 52. However, social-emotional (DQ = 80) and adaptive-behavior scores (DQ = 84) were relatively preserved.

Her parents had noticed sudden tonic extension movements of both arms one week previously, and characteristically, spasms were prominent during awakening or while falling asleep and consisted of 20–30 spasms per cluster. There was no family history of epilepsy or neurodevelopmental/neuropsychiatric disorders.

**Figure 1:**
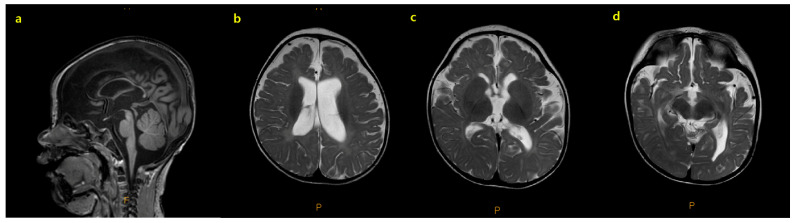
Brain magnetic resonance image(MRI) findings of the patient. Brain MRI findings of the patient. Dysmorphic thin corpus callosum was observed in the T1 weighted sagittal image (**a**). The diffuse cerebral atrophy, characterized by prominent extra axial space, prominent cerebral sulci with decreased volume, and delayed myelination of white matter were also evident (**b**–**d**).

**Figure 2:**
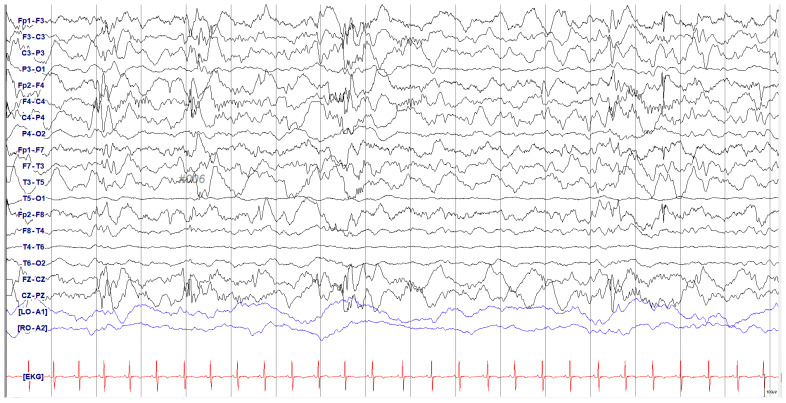
Electrographic encephalograph(EEG) of the patient. Electrographic encephalography recording of the patient with spasm-like movement. Background activities were of relatively high amplitude with multifocal spikes or polyspike discharges consistent with hypsarrhythmia.

An initial assessment was undertaken to identify the seizure etiology and her milestone delays. Brain MRI showed diffuse cerebral atrophy, characterized by enlarged extra-axial cerebrospinal fluid spaces, prominent cerebral sulci, and a thin corpus callosum (Figure 1). Ictal electroencephalography (EEG) was captured during an epileptic seizure event and featured diffuse spike and polyspike and wave discharges followed by an electro-decremental period. Inter-ictal EEG showed multifocal spike discharges with high-amplitude background activity diagnostic of hypsarrhythmia and infantile epileptic spasms syndrome (Figure 2). The patient was treated with vigabatrin starting from 50 mg/kg/day and titrated to 150 mg/kg/day. However, due to persistent epileptic spasms, further treatment with high-dose steroids was initiated. Transthoracic echocardiography and abdominal ultrasonography findings were unremarkable.

**Figure 3:**
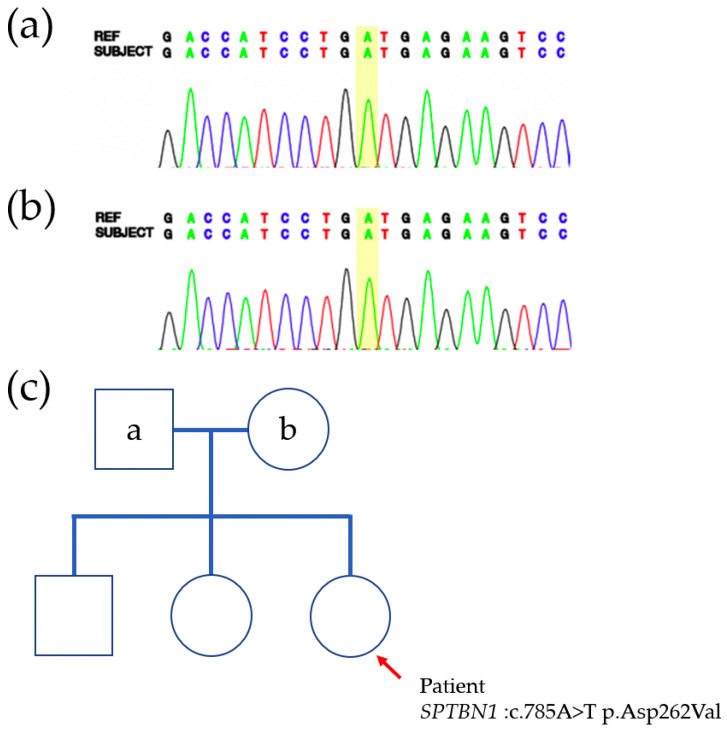
The pedigree of the patient and the whole exome sequencing/Sanger sequencing result. (**a**) Sanger sequencing of *SPTBN1* gene of father (wild type). (**b**) Sanger sequencing of *SPTBN1* gene of mother (wild type). (**c**) Pedigree of the family and the result of whole exome sequencing of the *SPTBN1* mutation in case patient.

Chromosomal Microarray Analysis (CMA) yielded normal results, while whole exome sequencing identified a de novo heterozygous variant in uncertain significance *SPTBN1* (c.785A>T; p.Asp262Val). Pedigree analysis using familial Sanger sequencing was also performed (Figure 3). According to ACMG classification, *SPTBN1* variant was reclassified with likely pathogenic variant. The allele frequency of the variant <0.01 in the gnomAD database in the gnomAD v4.0.0 dataset. In silico tool predictions suggest the damaging effect of the variant on gene or gene product [REVEL: 0.94 (≥0.6, sensitivity 0.68 and specificity 0.92); 3Cnet: 0.99 (≥0.6, sensitivity 0.72 and precision 0.9)].

## 4. Discussion

This is the first reported pediatric case of an IESS patient with a de novo *SPTBN1*, c.785A>T (p.Asp262Val) mutation. Patients with *SPTBN1* mutations have been originally reported in patients with ASDs [4], though it has subsequently been reported in patients with variable neuropsychiatric disorders, such as epilepsy, intellectual disability, speech disorders, attention-deficit/hyperactivity disorder [3], OMIM#182790.

IESS is one of the devastating epileptic encephalopathies which has a broader etiological spectrum—including structural, genetic, metabolic, or infectious causes—and a more variable developmental outcome depending on early diagnosis and treatment. Under the development of epilepsy genetics, over 28 copy number variants and 70 single gene pathogenic variants related to IESS have been discovered to date [5]. Genetic variants comprise chromosomal disorders (e.g., trisomy21), single gene disorders (*TSC1*, *TSC2*, *CDKL5*, *ARX*, *KCNQ2*, *STXBP1* and *SCN2A*), trinucleotide repeat disorders, mitochondrial disorders, and possible candidate genes are reported [5]. Like other single gene disorders, the *SPTBN1* mutation could be a candidate gene which is associated with multiple cellular processes related to neuronal development and the generation of action potentials.

Spectrins are crucial cytoskeletal proteins that are ubiquitously expressed in the nervous system. Pathogenic variants in genes encoding neuronal spectrins, such as *SPTAN1*, *SPTBN1*, *SPTBN2*, and *SPTBN4*, are known to be associated with various neurodevelopmental disorders [2,6,7,8]. Among genes of the spectrin family, *SPTBN1* encodes neuronal βII-spectrin, which is the most abundant β-spectrin in the brain and forms αII-spectrin tetramers, which intercalate F-actin rings to build a sub-membranous periodic skeleton (MPS) [9]. Furthermore, a cytosolic pool of βII-spectrin promotes bidirectional axonal organelle transport [10].

Animal models deficient in βII-spectrin exhibit cortical disorganization, developmental delays, and behavioral deficits, whereas homozygous knockouts result in early postnatal lethality. Heterozygous models display milder yet significant phenotypes, suggesting that heterozygous *SPTBN1* variants may similarly impair neural development and function [3,10].

βII-spectrin consists of two calponin homology (CH1 and CH2) domains, 14–17 SR domains, and a PH domain [1,2]. Variants frequently cluster within the CH domains, particularly the CH2 domain, which exhibits a higher degree of missense constraint (ExAC v.10) and underscores its functional importance [11].

**Figure 4:**
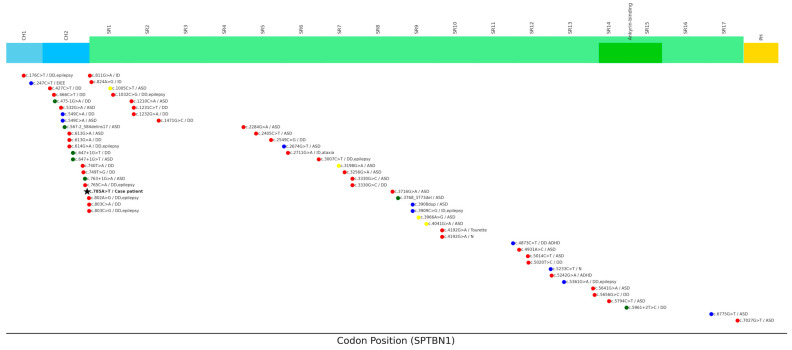
Schematic representation of functional domains of βII-spectrin. CH1 (sky blue), CH2, (blue), SR (green), PH (yellow). The locations of *SPTBN1* variants are indicated. Missense variants (red). Stop-gained /Frameshift variants (blue), Splice-site variants (dark green), Synonymous variants (yellow), other variants (black). CH1, calponin homology domain 1; CH2, calponin homology domain 2; SR, spectrin repeat; PH, pleckstrin homology domain; ASD, autism spectrum disorder; DD, delayed development; ADHD, attention deficit hyperactivity disorder; ID, intellectual disability; EIEE, early infantile epileptic encephalopathy; N, Not known. Our case’s patient was also marked with a black star.

Clinical heterogeneity was reported for individuals with a *SPTBN1* mutation as they exhibit variable degrees of neuropsychiatric disorientation. In addition, 14 research studies have reported associations between *SPTBN1* mutations and neuropsychiatric disorders; to date, 60 variants have been reported [1,3,4,12,13,14,15,16,17,18,19,20,21,22]. A mutation map is shown in Figure 4. Supplementary Table S1 shows that 91% (55/60) of affected individuals present with developmental delay, intellectual disability, and/or ASDs. Epilepsy has been reported in 15% (9/60) of cases, and epilepsy-associated mutations were found to favor CH domains (31.8%, 7/22) over SR domains (10.3%, 4/39). Notably, one other patient harbored a stop-gain mutation in the CH2 domain (c.247C>T; p.Arg83Ter), which was associated with a severe epileptic encephalopathy, presenting as early infantile epileptic encephalopathy (EIEE).

The association between spectrin mutations and epileptic encephalopathy has been reported only in cases involving *SPTAN1* mutations which encode αII-spectrin. The pathogenic mechanism of these mutations involves the disruption of αII-spectrin interactions with βII-spectrin, leading to the mislocalization of voltage-gated sodium channels and subsequent epileptic activity [23]. Conversely, our patient harbored a mutation in the CH2 domain of βII-spectrin which interacts with αII-spectrin. This mutation may disrupt the formation of α/β-spectrin tetramers and compromise cytoskeletal integrity and thereby might contribute to the development of severe epileptic phenotypes.

Moreover, as previously reported in an animal model [10], our patient demonstrated the phenotypical characteristics of corpus callosal hypoplasia, decreased white matter volume, and atrophic brain parenchyma. In addition, previously reported *SPTBN1*-associated epilepsy may be less severe and/or less penetrant than that associated with other spectrin mutations [3,22]. Our patient had IESS suggesting that the CH2 domain may play an important role in neurodevelopmental disorders, as well as epileptic seizure generation, by interacting with the αII domain.

However, this study has several limitations. As a single case report, the functional validation of the identified *SPTBN1* variant and its specific domain-level were not conducted. Although the variant was de novo, predicted to be deleterious, and reclassified as likely pathogenic according to ACMG guidelines, confirmatory Sanger sequencing was not performed to validate the mutation in the proband. Moreover, in the reported 14 pieces of literature addressed, the patients who were enrolled in the studies were analyzed in retrospective manner; specific types of epilepsy, severity, and seizure type were not reported.

This study demonstrates that the genotype–phenotype relationship of *SPTBN1* should be expanded and that rare mutations, like this one in the SPTBN1, can cause a severe form of epilepsy, IESS. This case report also suggests that structural abnormalities caused by minor genetic mutations can alter functionally critical cascades. In future studies, domain-specific pathogenicity within *SPTBN1* warrants functional studies at the cellular and animal levels to elucidate the mechanistic basis of βII-spectrin-related disorders.

## Data Availability

Data are contained within the article and supplementary materials.

## Supplementary Materials

The following supporting information can be downloaded at: https://www.mdpi.com/article/10.3390/genes16080904/s1, Supplemental Table S1: Previously reported genetic variants.

## Abbreviations

ADHD	Attention deficit hyperactivity disorder
ASD	Autism spectrum disorder
CH	Calponin homology
CMA	Chromosomal Microarray Analysis
DD	Delayed development
DQ	Developmental quotient
EEG	Electroencephalography
EIEE	Early infantile epileptic encephalopathy
HC	Head circumference
ID	Intellectual disability
IESS	Infantile epilepsy spasm syndrome
PH	Pleckstrin homology domain
SR	Spectrin repeat

## References

[1] Liem R.K. (2016). Cytoskeletal Integrators: The Spectrin Superfamily. Cold Spring Harb. Perspect. Biol..

[2] Lorenzo D.N., Edwards R.J., Slavutsky A.L. (2023). Spectrins: Molecular organizers and targets of neurological disorders. Nat. Rev. Neurosci..

[3] Cousin M.A., Creighton B.A., Breau K.A., Spillmann R.C., Torti E., Dontu S., Tripathi S., Ajit D., Edwards R.J., Afriyie S. (2021). Pathogenic SPTBN1 variants cause an autosomal dominant neurodevelopmental syndrome. Nat. Genet..

[4] Iossifov I., O’Roak B.J., Sanders S.J., Ronemus M., Krumm N., Levy D., Stessman H.A., Witherspoon K.T., Vives L., Patterson K.E. (2014). The contribution of de novo coding mutations to autism spectrum disorder. Nature.

[5] Snyder H.E., Jain P., RamachandranNair R., Jones K.C., Whitney R. (2024). Genetic Advancements in Infantile Epileptic Spasms Syndrome and Opportunities for Precision Medicine. Genes.

[6] Tohyama J., Nakashima M., Nabatame S., Gaik-Siew C.n., Miyata R., Rener-Primec Z., Kato M., Matsumoto N., Saitsu H. (2015). SPTAN1 encephalopathy: Distinct phenotypes and genotypes. J. Hum. Genet..

[7] Wang C.C., Ortiz-González X.R., Yum S.W., Gill S.M., White A., Kelter E., Seaver L.H., Lee S., Wiley G., Gaffney P.M. (2018). βIV Spectrinopathies Cause Profound Intellectual Disability, Congenital Hypotonia, and Motor Axonal Neuropathy. Am. J. Hum. Genet..

[8] Ikeda Y., Dick K.A., Weatherspoon M.R., Gincel D., Armbrust K.R., Dalton J.C., Stevanin G., Dürr A., Zühlke C., Bürk K. (2006). Spectrin mutations cause spinocerebellar ataxia type 5. Nat. Genet..

[9] Xu K., Zhong G., Zhuang X. (2013). Actin, spectrin, and associated proteins form a periodic cytoskeletal structure in axons. Science.

[10] Lorenzo D.N., Badea A., Zhou R., Mohler P.J., Zhuang X., Bennett V. (2019). βII-spectrin promotes mouse brain connectivity through stabilizing axonal plasma membranes and enabling axonal organelle transport. Proc. Natl. Acad. Sci. USA.

[11] Lek M., Karczewski K.J., Minikel E.V., Samocha K.E., Banks E., Fennell T., O’Donnell-Luria A.H., Ware J.S., Hill A.J., Cummings B.B. (2016). Analysis of protein-coding genetic variation in 60,706 humans. Nature.

[12] Cirnigliaro M., Chang T.S., Arteaga S.A., Pérez-Cano L., Ruzzo E.K., Gordon A., Bicks L.K., Jung J.Y., Lowe J.K., Wall D.P. (2023). The contributions of rare inherited and polygenic risk to ASD in multiplex families. Proc. Natl. Acad. Sci. USA.

[13] O’Connell M., Harstad E., Aites J., Hayes K., Arnett A.B., Scotellaro J., Patel S., Brewster S.J., Barbaresi W., Doan R.N. (2025). Diverse clinical presentation of SPTBN1 variants: Complex versus primary attention-deficit/hyperactivity disorder. Am. J. Med. Genet. A.

[14] Sandoval-Talamantes A.K., Tenorio-Castaño J.A., Santos-Simarro F., Adán C., Fernández-Elvira M., García-Fernández L., Muñoz Y., Lapunzina P., Nevado J. (2023). NGS Custom Panel Implementation in Patients with Non-Syndromic Autism Spectrum Disorders in the Clinical Routine of a Tertiary Hospital. Genes.

[15] Satterstrom F.K., Kosmicki J.A., Wang J., Breen M.S., De Rubeis S., An J.Y., Peng M., Collins R., Grove J., Klei L. (2020). Large-Scale Exome Sequencing Study Implicates Both Developmental and Functional Changes in the Neurobiology of Autism. Cell.

[16] Schmidt A., Danyel M., Grundmann K., Brunet T., Klinkhammer H., Hsieh T.C., Engels H., Peters S., Knaus A., Moosa S. (2024). Next-generation phenotyping integrated in a national framework for patients with ultrarare disorders improves genetic diagnostics and yields new molecular findings. Nat. Genet..

[17] Tuncay I.O., Parmalee N.L., Khalil R., Kaur K., Kumar A., Jimale M., Howe J.L., Goodspeed K., Evans P., Alzghoul L. (2022). Analysis of recent shared ancestry in a familial cohort identifies coding and noncoding autism spectrum disorder variants. NPJ Genom. Med..

[18] Woodbury-Smith M., Lamoureux S., Begum G., Nassir N., Akter H., O’Rielly D.D., Rahman P., Wintle R.F., Scherer S.W., Uddin M. (2022). Mutational Landscape of Autism Spectrum Disorder Brain Tissue. Genes.

[19] Wu R., Li X., Meng Z., Li P., He Z., Liang L. (2024). Phenotypic and genetic analysis of children with unexplained neurodevelopmental delay and neurodevelopmental comorbidities in a Chinese cohort using trio-based whole-exome sequencing. Orphanet J. Rare Dis..

[20] Zhou X., Feliciano P., Shu C., Wang T., Astrovskaya I., Hall J.B., Obiajulu J.U., Wright J.R., Murali S.C., Xu S.X. (2022). Integrating de novo and inherited variants in 42,607 autism cases identifies mutations in new moderate-risk genes. Nat. Genet..

[21] Willsey A.J., Fernandez T.V., Yu D., King R.A., Dietrich A., Xing J., Sanders S.J., Mandell J.D., Huang A.Y., Richer P. (2017). De Novo Coding Variants Are Strongly Associated with Tourette Disorder. Neuron.

[22] Rosenfeld J.A., Xiao R., Bekheirnia M.R., Kanani F., Parker M.J., Koenig M.K., van Haeringen A., Ruivenkamp C., Rosmaninho-Salgado J., Almeida P.M. (2021). Heterozygous variants in SPTBN1 cause intellectual disability and autism. Am. J. Med. Genet. A.

[23] Saitsu H., Tohyama J., Kumada T., Egawa K., Hamada K., Okada I., Mizuguchi T., Osaka H., Miyata R., Furukawa T. (2010). Dominant-negative mutations in alpha-II spectrin cause West syndrome with severe cerebral hypomyelination, spastic quadriplegia, and developmental delay. Am. J. Hum. Genet..

